# Effect of additional equipotent fentanyl or sufentanil administration on recovery profiles during propofol-remifentanil–based anaesthesia in patients undergoing gynaecologic laparoscopic surgery: a randomized clinical trial

**DOI:** 10.1186/s12871-022-01671-z

**Published:** 2022-04-29

**Authors:** Chunyuan Zhang, Ding Huang, Wei Zeng, Jian Ma, Ping Li, Qichang Jian, Jiamin Huang, Huanlong Xie

**Affiliations:** Department of Anesthesiology, Affiliated Boai Hospital of Zhongshan, Southern Medical University, No. 6 Chenggui Road, East District, Zhongshan, 528400 Guangdong People’s Republic of China

**Keywords:** Sufentanil, Fentanyl, Tracheal intubation, Emergence, Equipotent doses, Gynaecologic laparoscopic surgery

## Abstract

**Background:**

In clinical practice, sufentanil has a stronger sedative effect on patients than fentanyl at equivalent doses. This study hypothesized that, at equivalent doses, patients undergoing gynaecologic laparoscopic surgery (GLS) receiving fentanyl would have an earlier emergence from anaesthesia (EA), a shorter time to extubation (TE), and a better degree of wakefulness. Therefore, this study evaluated the effects of equipotent doses of fentanyl and sufentanil on the quality of emergence in patients undergoing GLS.

**Methods:**

One hundred seven patients scheduled for GLS under general anaesthesia were randomly divided into two groups and were induced with 0.35 µg/kg sufentanil (Group S; *n* = 55) or 3.5 µg/kg fentanyl (Group F; *n* = 52). When the GLS was almost over, the patient's abdominal cavity was flushed with warm saline, and 5 µg of sufentanil or 50 µg of fentanyl in a double-blind manner was intravenously injected into the patients. The primary outcomes of the study included EA, TE, the rate of leaving the surgical bed voluntarily and the incidence of endotracheal tube tolerance. The Ramsay Sedation Scale (RSS), and Verbal Rating Scale (VRS) scores at 15 and 30 min in the postanaesthesia care unit (PACU), as well as other adverse events, including nausea and vomiting, itching, delirium, dizziness, chills, and respiratory depression (SpO_2_ < 95%) in the PACU, were evaluated as secondary outcomes.

**Results:**

There were no statistically significant dissimilarities between the two groups with respect to baseline characteristics. For recovery, the EA (9.0 ± 4.8 min vs. 8.9 ± 3.0 min; *P* = 0. 146), TE (9.5 ± 4.7 min vs. 9.0 ± 3.0 min; *P* = 0.135), rate of leaving the surgical bed voluntarily (31.18% vs. 38.46%; *P* = 0.976), and incidence of endotracheal tube tolerance (94.55% vs. 96.15%; *P* = 0.694) were not significantly different between the two groups. In the PACU, the 15-min RSS score (2.07 ± 0.38 vs. 2.15 ± 0.36; *P* = 0.125), the 30-min RSS score (2.02 ± 0.13 vs. 2.04 ± 0.19; *P* = 0.207), the 15-min VRS score (0.50 ± 0.57 vs. 0.67 ± 0.55; *P* = 0.295), and the 30-min VRS score (0.45 ± 0.50 vs. 0.75 ± 0.52; *P* = 0.102) were not significantly different between Groups S and F. No adverse events, such as nausea, vomiting, pruritus, delirium, and tremors, occurred in either group. The rates of respiratory depression (1.82% vs. 1.92%; *P* = 0.968) and dizziness (0.00% vs. 4.85%; *P* = 0.142) were not different between Groups S and F in the PACU.

**Conclusions:**

The majority of patients scheduled for GLS were able to rapidly and smoothly emerge from anaesthesia. After surgery, similar outcomes, including EA, TE, the incidence of endotracheal tube tolerance, the rate of leaving the surgical bed voluntarily, RSS scores, VRS scores, and adverse events in the PACU, were achieved for the patients between the two anaesthetic protocols.

## Background

Laparoscopic surgery has the characteristics of minimal trauma, accuracy, and rapid recovery [1]. With good results and prognosis in the treatment of various gynaecologic diseases, gynaecologic laparoscopic surgery (GLS) has gradually replaced traditional open surgery as the main surgical method for women [2]. Patients undergoing laparoscopic surgery require a CO2 pneumoperitoneum and the head-down position, which may make patients more susceptible to poor quality of emergence and other adverse outcomes, such as hypoxia, vomiting, nausea and postoperative infection [3–5]. Therefore, it is essential to choose the optimal general anaesthesia for patients undergoing laparoscopic surgery [3–6]. However, there is no one drug that can meet all of the requirements for anaesthesia (i.e., unconsciousness, analgesia, amnesia, and muscle relaxation) [5, 6]. The administration of several different agents is needed to enhance perioperative safety and the quality of recovery, including sedation (propofol), analgesia (sufentanil and fentanyl), and muscle relaxation (cis-atracurium) [5, 6]. To increase perioperative safety and the quality of recovery, opioid analgesics are required [7]. Fentanyl and sufentanil are potent opioid analgesics that are frequently used in the clinical setting because of their ability to reduce surgical trauma, reduce the stress response, and maintain perioperative haemodynamic stability [7–9]. Previous studies have also shown that propofol or remifentanil maintenance is safe for use in GLS patients [5, 6, 10].

One of the primary goals following ambulatory surgery should be postoperative quality of recovery [11]. It is well established that total intravenous anaesthesia with opioids improves the postoperative quality of recovery following ambulatory surgery. Opioids, on the other hand, have been linked to an increased frequency of postoperative complications, which can impair the postoperative quality of recovery [7]. The minimum effective plasma concentration for fentanyl is 0.23–0.99 ng/mL [12] and 0.025–0.05 ng/mL for sufentanil [13]. Clinically, sufentanil has a stronger sedative effect on patients than fentanyl at equivalent doses as reported [14–16].

Therefore, this study hypothesized that if additional equivalent doses of sufentanil or fentanyl were given to GLS patients during propofol-remifentanil-based anaesthesia, patients in the fentanyl group would have an earlier emergence from anaesthesia (EA), a shorter time for time-to-extubation (TE), and a better degree of wakefulness (five minutes after extubation, more patients able to actively move from the operating bed to the transfer bed). The objective of this study was to compare the effect of additional equipotent doses of sufentanil or fentanyl on the quality of emergence in patients undergoing GLS during propofol-remifentanil-based anaesthesia.

## Methods

The Ethics Committee of the Affiliated Boai Hospital of Zhongshan (Southern Medical University) gave ethical permission for the study on April 26, 2020 (Ethical Committee No. KY-2020–004-15; Zhongshan, Guangdong, China). All patients gave written informed consent before participation in this study, and all procedures of this study followed the tenets of the Helsinki Declaration. The Chinese Clinical Trial Registry (https://www.chictr.org.cn/abouten.aspx) has been notified about this trial (ChiCTR2000032396, 27/04/2020).

### Study design

From April 26, 2020, to October 31, 2020, we recruited 107 patients (18–50 years of age) with gynaecologic disease at the Boai Hospital of Zhongshan, Southern Medical University. The first patient was enrolled on April 28, 2020. The patients had American Society of Anesthesiologists (ASA) physical status I or II for elective GLS.

The patients were randomized to two groups using a computer-generated random number table to receive either fentanyl (Group F, *n* = 52) or sufentanil (Group S, *n* = 55). Group assignments were sealed in sequentially numbered opaque envelopes that were opened by an investigator not involved in data collection. The anaesthesiologist was the only person informed of the randomization results and was not involved in the postoperative management or data evaluation.

The exclusion criteria were as follows: age < 18 years or > 65 years; alcohol or drug abuse; opioid dependence; chronic pain; pain medication prior to surgery; sedation received within 24 h prior to the study; infection at the injection site; impaired kidney or liver function; the presence of a gastric tube; a history of asthma, chronic cough, and smoking; respiratory infection within 2 weeks of study enrolment; “difficult” airway; allergy to nondepolarizing muscle relaxants and opioids; myasthenia gravis; schizophrenia; severe depression; severe anaemia; coagulopathy; New York Heart Association class > II; systolic blood pressure ≤ 90 mmHg or diastolic blood pressure ≥ 100 mmHg; bronchodilator or corticosteroid use; and psychiatric sicknesses that would interfere with pain assessment.

### Patient management

Patients were not given any medications preoperatively, and they received no solid food for 6 h and no water for 2 h. After the patient entered the operating room, Ringer's solution was infused at a rate of 10 ml/kg/h, and the electrocardiogram (ECG), blood pressure, heart rate (HR), pulse oximetry (SpO_2_), and end-tidal CO_2_ (ETCO_2_) were monitored. The depth of anaesthesia was monitored by Quatro (Narcotrend; MT Monitortechnik GmbH & Co., KG, Bad Bramstedt, Germany).

### Anaesthesia regimens

After preoxygenation for 3 min, anaesthesia was induced with 2.5 mg/kg of propofol intravenously over 10–20 s. This was followed by an intravenous injection of 3.5 µg/kg fentanyl (Group F) or 0.35 µg/kg sufentanil (Group S) over 20–30 s. One minute later, 0.1 mg/kg cis-atracurium was injected intravenously over 10–20 s, and 2 min later, endotracheal intubation was performed under visual laryngoscopy guidance (tracheal tube #7.0). The maintenance dose of anaesthesia was 6–10 mg/kg/h for propofol and 0.1–0.3 µg/kg/min for remifentanil. Typically, patients were induced on higher doses and subsequently titrated according to the depth of anaesthesia, which was maintained between E1 and D2 (20–46). Ventilation was mechanically regulated with a 60% oxygen-air gas combination to maintain an ETCO_2_ concentration of 35–40 mmHg. During the anaesthetic and recovery phases, haemodynamic stability was ensured. When hypotension (SBP < 90 mmHg) occurred, ephedrine (6 mg) was administered, while hypertension (SBP > 140 mmHg) was treated with urapidil (5 mg). When bradycardia (HR < 50 beats/min) occurred, atropine (0.5 mg) was given intravenously, whereas tachycardia (HR > 100 beats/min) was treated with esmolol (10 mg). When the GLS was almost complete, the patient's abdominal cavity was flushed with warm saline, propofol was discontinued, and then remifentanil was discontinued during suturing. Fentanyl (50 µg) or sufentanil (5 µg) and topiramisone (5 mg) were administered intravenously in divided doses in a double-blind manner. The doses of propofol, remifentanil, fentanyl, and sufentanil were recorded. The patients were extubated when adequate voluntary ventilation and response to verbal commands were established.

### Evaluation indices

The time of EA was the time from completion of the procedure to positioning (recalling one’s name and date of birth). During emergence, the incidence of endotracheal tube tolerance, EA, and TE were recorded blinded by the same anaesthesiologist. The rate of leaving the surgical bed voluntarily was also recorded. “Leaving the surgical bed voluntarily” was defined as being able to voluntarily move from the operating bed to the transfer bed with the assistance of ≤ 2 medical workers within 5 min after the tracheal tube was removed. Thereafter, the patients were transferred directly to the postanaesthesia care unit (PACU) for a stay of 30 min, where an independent blinded observer further recorded the 15- and 30-min Ramsay Sedation Scale (RSS) and Verbal Rating Scale (VRS) scores, as well as side effects, such as nausea and vomiting, itching, delirium, dizziness, chills, and respiratory depression (SpO_2_ < 95%). Postanaesthesia recovery was scored using the Steward Recovery Score (SRS) to determine eligibility for PACU discharge. Steward recovery scores were as follows: (i) level of consciousness (2 points: fully awake; 1 point: response to stimulation; 0 points: no response to stimulation); (ii) degree of airway patency (2 points: coughing according to an order; 1 point: maintaining airway patency without support; 0 points: supported respiration); and (iii) physical activity (2 points: conscious activity; 1 point: unconscious activity; 0 points: no limb activity). The criterion for discharge from the PACU was defined as an SRS > 4.

### Statistical analysis

In the 2 groups of 30 patients each, we conducted a preliminary study using the same procedure as that in the current investigation. Those study findings were never made public. Two patients (5%) in Group F and one patient (3%) in Group S had a choked cough response to endotracheal intubation. With a power of 80%, 2-sided α = 0.05 and β = 0.1, suggesting that 45 patients were required per group. To account for possible dropouts before the start of the study, we decided that 50 patients would be enrolled in each group. The equality of variances was analysed using Levene's test. Proportional data were analysed using a chi-square test. A value of *P* < 0.05 was considered statistically significant. Analyses were performed with IBM SPSS Statistics (version 24.0; IBM Corp., Armonk, NY, USA).

## Results

The two groups were comparable with respect to age, weight, ASA I/II, types of surgery, blood loss, urine volume, and infusion volume (all *P* > 0.05; Table 1). There were no significant differences between the two groups at baseline. The total doses of intraoperative anaesthetics and the operative time, including the total doses of sufentanil (19.57 ± 2.35 μg), fentanyl (192.4 ± 23.45 μg), remifentanil (0.8 ± 0.4 vs. 0.8 ± 1.4 mg), propofol (587.7 ± 230.8 vs. 487.0 ± 200.9 mg), and duration of surgery (70.9 ± 26.6 vs. 57.4 ± 27.7 min), were well matched between Groups F and S (all *P* > 0.05; Table 2). Although the propofol dosage and operative time were higher in Group S than in Group F, there was no statistically significant difference between the two groups. No notable haemodynamic differences were observed between the two groups at baseline.

**Table 1:** Patient demographic characteristics (mean ± SD)

Characteristic	Group S (*n* = 55)	Group F (*n* = 52)	*P* value
Age (years)	33.3 ± 5.9	32.1 ± 6.4	0.564
Weight (kg)	55.9 ± 6.7	54.4 ± 6.7	0.963
ASA physical status (I/II)	29/26	28/24	0.897
Types of surgery			
Ovarian cystectomy	35	32	0.893
Adnexectomy	12	11	0.912
Salpingectomy	8	9	0.923
Bleeding volume (ml)	34.8 ± 31.9	31.4 ± 52.8	0.208
Urine volume (ml)	146.7 ± 120.1	100.0 ± 113.8	0.845
Infusion volume (ml)	806.4 ± 221.5	788.5 ± 195.4	0.100

**Table 2:** Characteristics of anaesthesia management (mean ± SD)

Characteristic	Group S (*n* = 55)	Group F (*n* = 52)	*P* value
Sufentanil total dose (μg)	19.57 ± 2.35	–	–
Fentanyl total dose (μg)	–	192.4 ± 23.45	–
Remifentanil total dose (mg)	0.8 ± 0.4	0.8 ± 1.4	0.362
Propofol total dose (mg)	587.7 ± 230.8	487.0 ± 200.9	0.110
Duration of surgery (min)	70.9 ± 26.6	57.4 ± 27.7	0.092

### Comparison of the characteristics of emergence from anaesthesia

The incidence of endotracheal tube tolerance (%), EA (min), TE (min), and the rate of leaving the surgical bed voluntarily (%) after surgery are shown for both groups in Table 3. The analysis showed that there were no significant differences in the recovery time from anaesthesia, including endotracheal tube tolerance (94.55% vs. 96.15%), EA (9.0 ± 4.8 vs. 8.9 ± 3.0 min), and TE (9.5 ± 4.7 vs. 9.0 ± 3.0 min), and the rate of leaving the surgical bed voluntarily (38.18% vs. 38.46%) between Groups S and F (all *P* > 0.05).

**Table 3:** Characteristics of emergence from anaesthesia (mean ± SD)

Characteristic	Group S (*n* = 55)	Group F (*n* = 52)	*P* value
Endotracheal tube tolerance (%)	52(94.55)	50(96.15)	0.694
Time to emergence (min)	9.0 ± 4.8	8.9 ± 3.0	0.146
Time to extubation (min)	9.5 ± 4.7	9.0 ± 3.0	0.135
Rate of leaving the surgical bed voluntarily, no. (%)	21(38.18)	20(38.46)	0.976

Endotracheal tube tolerance was defined as the patient’s ability to first tolerate endotracheal intubation and then complete the commanded eye opening

### Comparison of the RSS and VRS scores in the PACU

We assessed the VRS scores and RSS scores at different stages in the PACU, including 15 and 30 min after extubation. We found that the VRS score in Group S (0.45 ± 0.50) at 30 min was lower than that in Group F (0.75 ± 0.52, *P* = 0.102; Table 4), but there was no statistical significance between Groups S and F. The RSS scores in Group S (2.07 ± 0.38 and 2.02 ± 0. 13) were not significantly higher than those in Group F (2.15 ± 0.36 and 2.04 ± 0.19, respectively) at each stage (all *P* > 0.05; Table 4).

**Table 4:** Characteristics of the Ramsay Sedation Scale (RSS) and Verbal Rating Scale (VRS) scores in the postanaesthesia care unit (PACU) (mean ± SD)

	15 min		30 min	
	RSS	VRS	RSS	VRS
Group S (*n* = 55)	2.07 ± 0.38	0.50 ± 0.57	2.02 ± 0.13	0.45 ± 0.50
Group F (*n* = 52)	2.15 ± 0.36	0.67 ± 0.55	2.04 ± 0.19	0.75 ± 0.52
*P* value	0.125	0.295	0.207	0.102

Ramsay Sedation Scale (*RSS*) and Verbal Rating Scale (*VRS*) scores at 15 and 30 min in the postanaesthesia care unit (*PACU*)

### Characteristics of adverse events in the PACU

We found no major adverse reactions in either Group F or Group S. There was one case (1.8%) of respiratory depression and two cases (3.9%) of dizziness in Group F, but there was no significant difference between the two groups (all *P* > 0.05; Table 5).

**Table 5:** Characteristics of adverse events in the postanaesthesia care unit (*N* = 107)

Characteristic	Group S (*n* = 55)	Group F (*n* = 52)	*P* value
Respiratory depression (SpO_2_ < 95%), n (%)	1(0.018)	1(0.019)	0.968
Nausea and vomiting, n (%)	0(0.00)	0(0.00)	–
Itching, n (%)	0(0.00)	0(0.00)	–
Delirium, n (%)	0(0.00)	0(0.00)	–
Dizziness, n (%)	0(0.00)	2(0.039)	0.142
Chills, n (%)	0(0.00)	0(0.00)	–

## Discussion

This prospective randomized controlled trial found that women receiving propofol-remifentanil-based anaesthesia and using additional sufentanil during total intravenous anaesthesia for GLS (Group S) had similar characteristics of emergence from anaesthesia (EA) as women who instead used additional fentanyl (Group F). Clinically, total intravenous anaesthesia is well tolerated by patients, is simple to perform [17, 18] and is a common procedure in GLS [19]. Therefore, our study chose total intravenous anaesthesia. Although they help to control postoperative pain, opioids also cause side effects [6–9], which may lead to a delay in healing and in the return to regular daily activities, as well as lower patient satisfaction [20]. Sufentanil has been reported to have a better analgesia effect [21] and shorter elimination half-life [22] than fentanyl. Thus, sufentanil is likely to provide more rapid extubation and improve the quality of emergence. The administration of fentanyl 10–15 min before the completion of surgery allows a faster recovery of regular breathing and early extubation [23]. Additionally, because remifentanil is a short-acting opioid, prophylactic administration of supplemental opioids provides effective transitional analgesia [24]. Considering that opioid analgesics are a primary determinant in reducing nociception and improving perioperative safety [25], fentanyl (50 µg) or sufentanil (5 µg) was infused intravenously before the completion of surgery in our study.

In a recent study, Son et al. [26] reported that in patients undergoing GLS, sufentanil administered before the conclusion of remifentanil anaesthesia reduced postoperative hyperalgesia and achieved haemodynamic stability during extubation without delaying recovery or worsening PONV. Sufentanil administration during remifentanil-based complete intravenous anaesthetic induction is linked to lower early postoperative opioid use [27]. Beloeil et al. [28] questioned the effect of an opioid-free balanced anaesthetic. Beloeil et al. [28] showed that individuals receiving opioid-free anaesthesia with dexmedetomidine experienced more significant side effects than those receiving remifentanil during elective intermediate or major noncardiac surgery. Compared to intraoperative opioid use, the elimination of opioid use was not as impressive [28].

When propofol and remifentanil were used simultaneously in both groups to maintain anaesthesia, the emergence characteristics between sufentanil and fentanyl gave a good indication of which opioid analgesic was more effective for GLS. To correctly compare the effects of sufentanil and fentanyl, propofol and remifentanil were used at similar doses in this study (*P* > 0.05). We found that there was no statistically significant difference with respect to the emergence characteristics, such as endotracheal tube tolerance, EA, TE, and the rate of leaving the surgical bed voluntarily.

These results suggest that sufentanil was as effective as fentanyl in improving the quality of emergence. Our findings are consistent with the findings of other studies [29–31]. Arun et al. [20] did not show the effect of opioids (fentanyl vs. sufentanil) in total intravenous anaesthesia on the TE for major spinal surgery, which is similar to our approach. It is possible that the TE may have been influenced more by the dosage of propofol than by the opioid [31]; however, there are few studies focusing on endotracheal tube tolerance with different opioids during emergence in the operating room, and there is a lack of information on intraoperative extubation. In this study, the endotracheal tube tolerance was good, and haemodynamic stability was achieved.

In addition, by comparing the data from the two groups, we detected no difference in postoperative outcomes in the PACU. These findings indicated that at equipotent doses, both sufentanil and fentanyl cause few postoperative adverse events and have similar sedative and analgesic effects. The incidence of respiratory depression and dizziness was very low, and we did not note opioid-related side effects [6–9], including nausea and vomiting, itching, delirium, and chills. No differences were observed between the two drugs, possibly because both drugs have potent analgesic effects in the fentanyl family and the combination of opioids reduced adverse effects caused by single use [32].

It should be pointed out that even though the dosage of propofol and the duration of surgery were higher in Group S than in Group F, there was no statistical significance between the two groups. In our study, dexmedetomidine, lidocaine, or tramadol [33] were not required to reduce cough or other responses related to tracheal irritation from the endotracheal tube. Recently, the concepts of rapid recovery from anaesthesia have become increasingly important. Our findings provide evidence that the use of fentanyl or sufentanil combined with propofol-remifentanil total intravenous anaesthesia during induction and at the end of warm saline peritoneal lavage before surgery completion is a substantial advantage to achieve a smooth and satisfactory emergence and improve the efficiency in GLS.

This study has several limitations. First, follow-up subsequent data (e.g., PONV, desaturation, hypotension, and dizziness) were not collected after the patient returned to the ward. Additional data would have helped us determine which opioid analgesic is more effective for GLS. Second, we did not define the basic procedure for extubation. Third, the applicability of our findings to patients under other anaesthetic management routines requires further validation. We cannot conclude that maintenance with isoflurane inhalation combined with remifentanil infusion would yield similar findings. Furthermore, the intravenous administration of sufentanil and fentanyl at the end of surgery might have induced mild sedation and smooth recovery because the effect of the opioids (sufentanil, fentanyl, remifentanil and even propofol) remained in the patients. Clinically, it might also be difficult to distinguish the effect of different opioids when administered at the end of surgery because patients might be too drowsy to express the VAS score exactly.

## Conclusion

In this study, the vast majority of patients scheduled for GLS were able to emerge quickly and without difficulty. After surgery, similar outcomes, including the incidence of endotracheal tube tolerance, EA, TE, rate of leaving the surgical bed voluntarily, RSS scores, VRS scores, and adverse events in the PACU, were achieved for the patients between the two anaesthetic protocols.

## Data Availability

The datasets analysed during the current study are available from the corresponding author on reasonable request.

## Abbreviations

GLS: Gynaecologic laparoscopic surgery
EA: Emergence from anaesthesia
TE: Time-to-extubation
RSS: Ramsay Sedation Scale
VRS: Verbal Rating Scale
PACU: Postanaesthesia care unit
